# Pan-cancer characterization of expression and clinical relevance of m^6^A-related tissue-elevated long non-coding RNAs

**DOI:** 10.1186/s12943-021-01324-8

**Published:** 2021-02-08

**Authors:** Kang Xu, Yangyang Cai, Mengying Zhang, Haozhe Zou, Zhenghong Chang, Donghao Li, Jing Bai, Juan Xu, Yongsheng Li

**Affiliations:** 1 Key Laboratory of Tropical Translational Medicine of Ministry of Education, College of Biomedical Information and Engineering, Hainan Medical University, Haikou, 571199 Hainan China; 2 College of Bioinformatics Science and Technology, Harbin Medical University, Harbin, 150081 China

## Main text

N^6^-methyladenosine (m^6^A) has become a critical internal RNA modification, and it plays important roles in the development and progression of cancer [1]. m^6^A has also been found in diverse non-coding RNAs, such as microRNAs and long noncoding RNAs (lncRNAs) [2]. LncRNAs comprise a large class of RNA transcripts and are critical regulators of gene expression. The regulatory effectiveness of lncRNAs is closely associated with spatial expression, whose dysregulation often influences cancer development and progression [3]. For these reasons, global characterization of lncRNA spatial expression across tissues or cancers could improve our understanding of lncRNA functions. Recently, LncRNA Spatial Atlas (LncSpA) and landscape of m^6^A have been proposed as valuable resources to understand lncRNA and m^6^A regulatory functions across different tissues [4, 5]. However, we still lack understanding of the distribution and functions of m^6^A modification in lncRNAs, particularly the tissue-elevated (TE) lncRNAs.

In this study, we aimed to systematically characterize the distribution and clinical relevance of m^6^A-related TE lncRNAs across tissues and cancer types. We found that TE lncRNAs were found to be regulated by m^6^A modification across tissues, particular brain tissues. We also investigated the correlation between expression of m^6^A regulators and TE lncRNAs, and found that numbers of m^6^A-related TE lncRNAs were associated with expression of m^6^A regulators. We assessed the clinical prognostic values of m^6^A-regulated TE lncRNAs. We identified several m^6^A-related TE lncRNAs as potentially useful markers for prognostic stratification. Our analysis highlights the importance of m^6^A modification in the regulation of lncRNA expression and helps bridge the knowledge gap between lncRNA expression and phenotypes.

## TE lncRNAs are associated with m^6^A modification across tissues

**Fig. 1:**
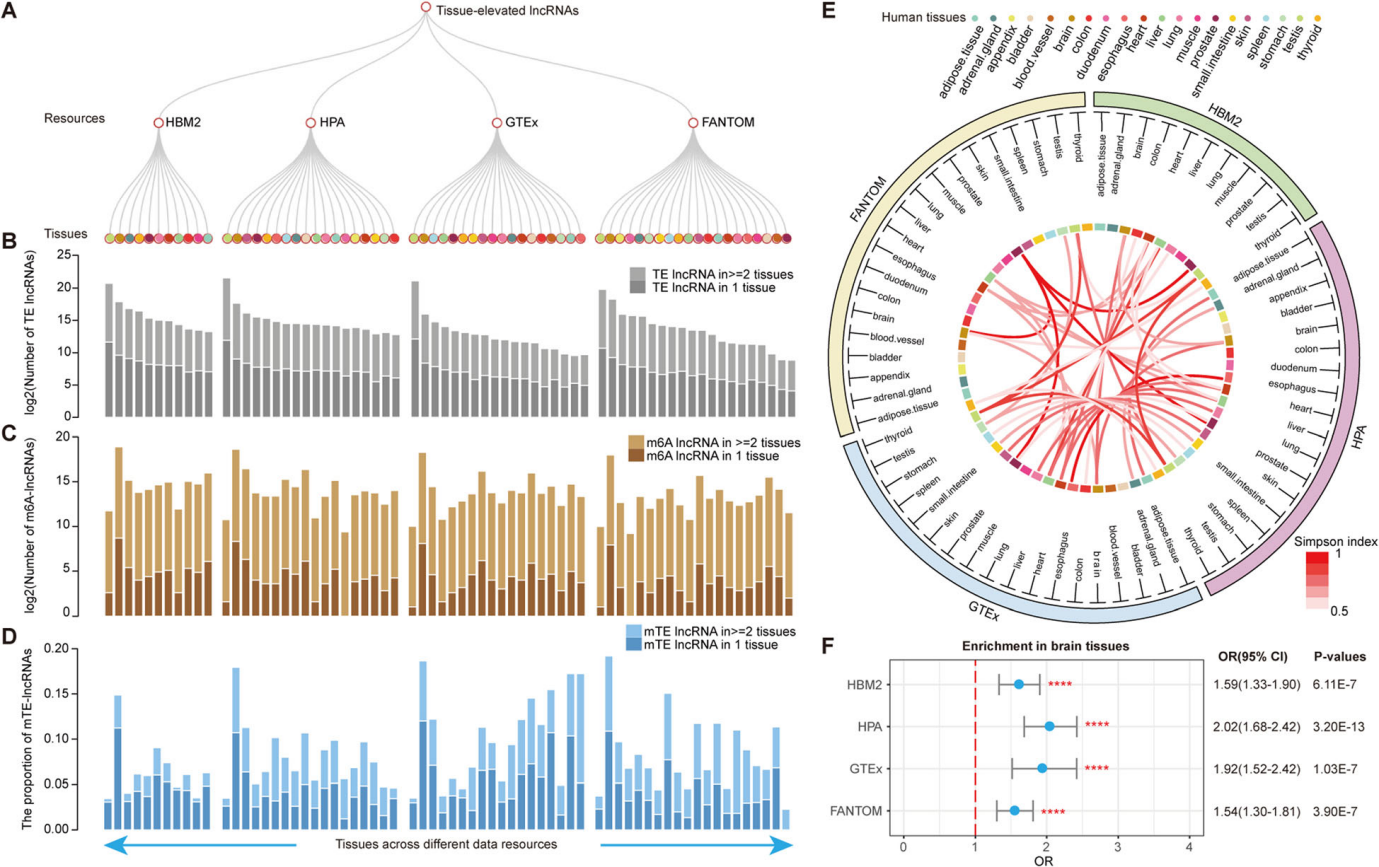
m^6^A-modified TE lncRNAs across human tissues. **a** Tissues from 4 data resources. **b** Bar plots showing the number of TE lncRNAs across tissues. Dark gray bars showing the number of TE lncRNAs observed only in representing tissues. **c** Number of m^6^A-modified lncRNAs across tissues. Dark brown bars showing the m^6^A-modified lncRNAs only in one tissue. **d** The proportion of m^6^A-modified TE lncRNAs across tissues. Dark blue bars represent the m^6^A-modified TE lncRNAs observed only in one tissue. **e** Circos plot showing the similarity between tissues based on overlap of m^6^A-modified TE lncRNAs. Simpson index was shown by inside links. **f** Odds ratios for comparison between m^6^A-modified TE lncRNAs and non-TE lncRNAs in brain tissues. Odds ratios and 95% confidence level, *p*-values were shown in the right side

We first retrieved the TE lncRNAs from 38 normal tissues from LncSpA in 4 data resources (Fig. 1a), including Human Body Map (HBM2.0), Human Protein Atlas (HPA), the Genotype-Tissue Expression (GTEx), and the Function Annotation Of The Mammalian Genome (FANTOM) project. In total, 9837, 13,337, 10,718, and 74,767 TE lncRNAs were obtained from GTEx, HPA, HBM2.0, and FANTOM5, respectively. Higher numbers of TE lncRNAs were found in tissues of the brain and testis tissues than in other tissues (Fig. 1b and Additional file 1: Table S1). Next, we mapped all the m^6^A modification peaks to lncRNAs and identified approximately 511–1600 lncRNAs regulated by m^6^A across tissues (Fig. 1c, Additional file 2: Figure S1 and Additional file 3: Table S2). We next assessed the proportion of m^6^A-modified TE lncRNAs among human tissues. We found that brain tissues had the highest proportion of TE lncRNAs with m^6^A modifications (Fig. 1d and Additional file 2: Figure S2). Approximately 14.89–19.20% TE lncRNAs were m^6^A-modified in brain tissues than in other tissues in four data resources. Although there were higher numbers of TE lncRNAs in testis tissues, the proportion of m^6^A-modified TE lncRNAs was small (Fig. 1d).

Next, we compared the overlap of m^6^A modified TE lncRNAs among tissues from different data resources. The Simpson index was calculated for two tissues from different sources. High correlations were observed for the same tissues across different sources (Fig. 1e), suggesting that m^6^A modified TE lncRNAs were conserved across different resources. To investigate potential tissue specificity of the m^6^A-modified TE lncRNAs, we calculated the percentage of m^6^A-modified TE lncRNAs and non-TE lncRNAs in each tissue. There were no significant differences observed for the two lncRNA categories for the most tissues, which is consistent with the observations in protein coding genes [5]. However, the proportion of m^6^A-modified TE lncRNAs is significantly higher than that of non-TE lncRNAs in brain tissues (Fig. 1f and Additional file 2: Figure S3). We explored the number of m^6^A peaks for lncRNAs across tissues. We found that the majority of m^6^A peaks in lncRNAs were in brain tissues (Additional file 2: Figure S4). Collectively, these results indicated that TE lncRNAs are associated with m^6^A modification across tissues and are more prone to be regulated by m^6^A in brain tissues than in other tissues.

## Co-expression network of TE lncRNAs and m^6^A regulators

The regulatory effects of m^6^A modification are primarily determined by regulators, including readers, writers, and erasers [6]. The extent to which variation in m^6^A modification of TE lncRNAs may be attributed to the expression of m^6^A regulators remains unknown. Thus, we next sought to analyze the correlation between the expressions of m^6^A-modified TE lncRNAs and regulators. In total, we identified 4862 correlations among 860 TE lncRNAs and 20 m^6^A regulators in 4 resources (Additional file 2: Figure S5A and Additional file 4: Table S3). Numbers of TE lncRNAs were associated with expression of m^6^A regulators in all four sources, including AC091878.1, LINC00854 and AC007879.5 (Additional file 2: Figure S5B). In contrast, we calculated the number of TE lncRNAs correlated with each m^6^A regulators. Higher numbers of TE lncRNAs were found to be correlated with the expression of IGF2BP1, METTL3 and VIRMA (Additional file 2: Figure S6).

Notably, we identified several TE lncRNA-regulator pairs that had been verified in literature. We took PVT1 as an example and found its expression to be significantly correlated with YTHDF2 (Additional file 2: Figure S5C, R = 0.64, *P* = 0.0003). Evidence has shown that YTHDF2 and PVT1 interact and that YTHDF2 plays critical roles in the stability of PVT1 [7]. Another example is SOX2-OT, which has been reported to play an oncogenic role in cancer. It was identified as a TE lncRNA in brain tissues from all four sources. We found its expression to be significantly closely correlated with HNRNPA2B1 (Additional file 2: Figure S5D, R = 0.56, *P* = 0.0005). It has been shown that SOX2-OT can regulate cancer proliferation and metastasis through the miR-146b-5p/HNRNPA2B1 pathway. We also found a significant correlation between KCNK15 − AS1 and ALKBH5 (Additional file 2: Figure S5E, R = 0.48, *P* = 0.0108). ALKBH5 had been demonstrated to inhibit cancer motility by demethylating lncRNA KCNK15-AS1 [8]. Together, all these results suggest that m^6^A modification of TE lncRNAs is partially regulated by the expression of m^6^A regulators.

## Association of m^6^A-modified TE lncRNAs with tumor prognosis

**Fig. 2:**
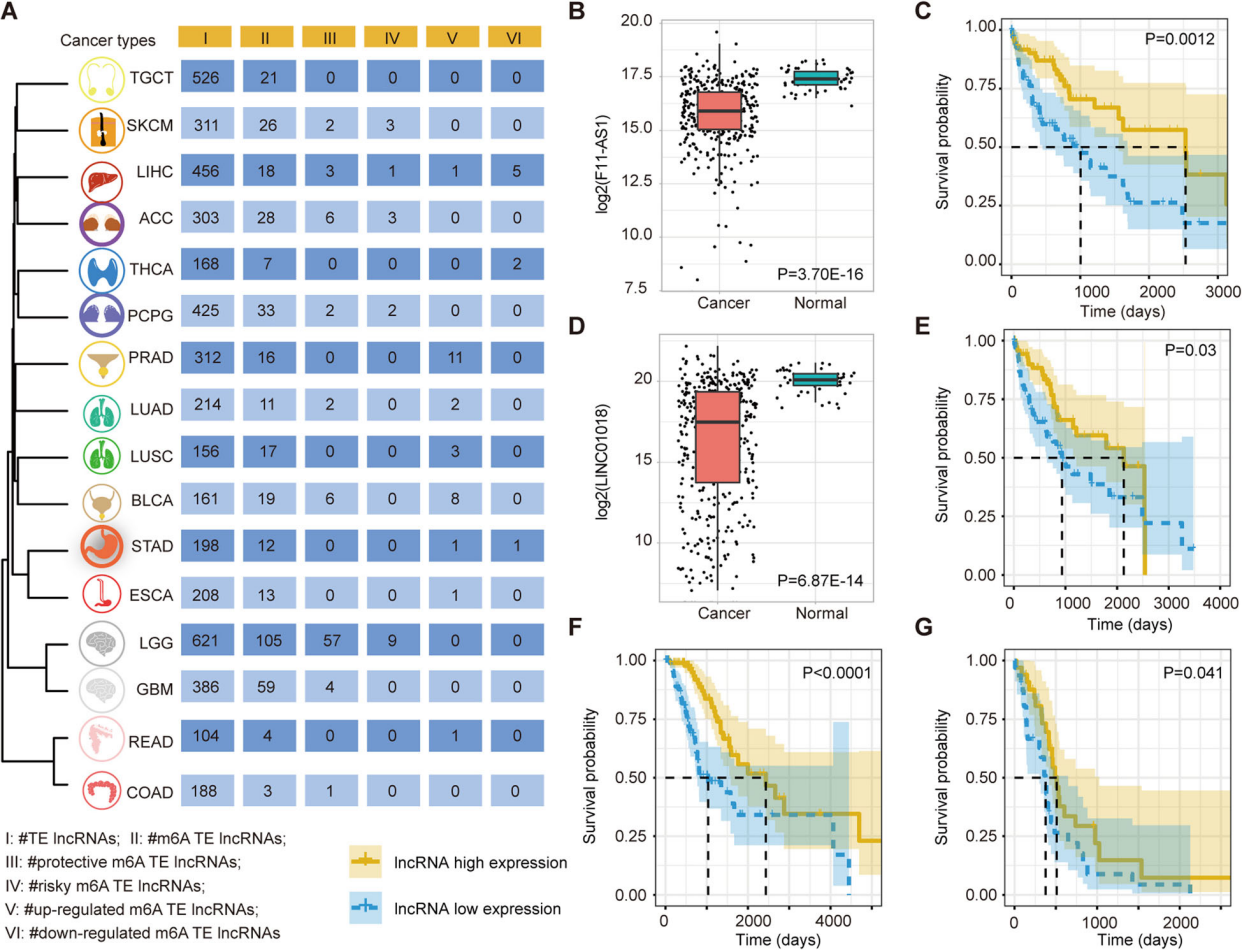
Clinical associations of m^6^A-modified TE lncRNAs across cancer types. **a** Heat map showing the number of TE lncRNA, m^6^A-modified TE lncRNAs, protective and risk lncRNAs, up- and down-regulated lncRNAs in each cancer. Cancer types were clustered together based on the overlap of TE lncRNAs. **b** Boxplots showing the expression of F11-AS1 in HCC patients and normal samples. **c** Kaplan-Meier plot for overall survival of HCC patients stratified by expression of F11-AS1. **d** Boxplots showing the expression of LINC01018 in HCC patients and normal samples. **e** Kaplan-Meier plot for overall survival of HCC patients stratified by expression of LINC01018. **f** Kaplan-Meier plot for overall survival of LGG patients stratified by expression of MIR325HG. **g** Kaplan-Meier plot for overall survival of GBM patients stratified by expression of MIR325HG

LncRNA has been identified as a biomarker suitable for the classification of cancer patients. We next investigated the relationship between expression of m^6^A modified TE lncRNAs and patient survival. We first manually mapped the m^6^A modification in human tissues to cancer types and identified 104–621 TE lncRNAs in 16 cancers (Fig. 2a). Cancers with similar tissue of origin were clustered together based on the overlap of TE lncRNAs, such as LGG and GBM, COAD, and READ. In addition, numbers of m^6^A-modified TE lncRNAs were identified across cancer types, ranging from 3 to 105 (Fig. 2a and Additional file 2: Figure S7).

We next explored the differences in survival between patients with high- and low-levels of lncRNA expression and identified 83 protective and 18 risky m^6^A-modified TE lncRNAs across cancer types (Fig. 2a and Additional file 5: Table S4). Moreover, we identified 28 m^6^A-modified TE lncRNAs that had significantly higher expression in cancer patients than in healthy controls and 8 m^6^A-modified TE lncRNAs that had significantly lower expression (Fig. 2a and Additional file 5: Table S4). There were two m^6^A-modified TE lncRNAs (F11-AS1 and LINC01018) showing significantly lower expression in hepatocellular carcinoma patients than in controls, and these lower expressions were associated with worse survival rates (Fig. 2b-e). F11-AS1 can inhibit HBV-related hepatocellular carcinoma progression by regulating NR1I3 via binding to microRNA-211-5p. LINC01018 has a novel tumor suppressor role in hepatocellular carcinoma by sponging miR-182-5p [9, 10]. We also found lower expression of m^6^A-modified MIR325HG to be correlated with worse patient survival in both LGG and GBM (Fig. 2f-g). These results suggest that these TE lncRNAs could be potentially tumor suppressors in cancer.

We next tried to determine the functions of F11-AS1, LINC01018 and MIR325HG. We performed Gene Set Enrichment Analysis (GSEA) on cancer patients. We found that these m^6^A-modified lncRNAs were involved in a number of cancer hallmark-related functions (Additional file 2: Figure S8 and Additional file 6: Table S5), such as DNA repair and epithelial mesenchymal transition pathways (Additional file 2: Figure S9). Taken together, all these results suggest a connection between m^6^A modified TE lncRNAs and the risk of diseases.

## Conclusions

We have shown the prevalence of m^6^A modification in TE lncRNAs across tissues and cancer types. The expression levels of m^6^A-modified TE lncRNAs were significantly closely associated with the activity of m^6^A regulators. Several studies have also shown that m^6^Am can regulate the expression of noncoding RNAs. Thus, it would also be interesting to integrate such m^6^A and m^6^Am data to identify potential lncRNA biomarkers in cancer. In summary, our work reveals the landscape of m^6^A-modified TE lncRNAs and provides a valuable resource for functional studies of m^6^A and lncRNA functions in the future.

## Supplementary Information

**Additional file 1: Table S1.** Number of TE lncRNAs, m^6^A-modified lncRNAs and the proportion of m^6^A-modified TE lncRNAs across tissues in four resources.

**Additional file 2: **Supplemental materials and methods, and supplemental figure S1-S9.**Figure S1.** Numbers of m^6^A-regulated lncRNAs across tissues. **Figure S2.** Numbers of m^6^A-regulated TE lncRNAs across tissues in four data resources. **Figure S3.** Distribution of odds ratios for comparison between TE lncRNAs and non-TE lncRNAs across tissues in four data resources. **Figure S4.** Number of m^6^A peaks correlated with TE lncRNAs in four resources. **Figure S5.**Co-expression between m^6^A regulators and m^6^A-modified TE lncRNAs. A, River plot showing the expression correlation between m^6^A modified TE lncRNAs and m^6^A regulators. B, Bar plots showing the number of m6A regulators correlated with each m6A modified TE lncRNA. Color indicated the different data resources. C-E, Scatter plots showing the correlation between the expression of lncRNAs and m^6^A regulators. C for PVT1 and YTHDF2; D for SOX2-OT and HNRNPA2B1; E for KCNK15-AS1 and ALKBH5. **Figure S6.** Numbers of TE lncRNAs correlated with m^6^A regulators. **Figure S7.** Number of m^6^A modified TE lncRNAs across cancer types. **Figure S8.** GSEA for m^6^A modified lncRNAs in HCC, LGG and GBM. A, F11-AS1 in HCC; B, LINC01018 in HCC; C, MIR325HG in LGG, D, MIR325HG in GBM. **Figure S9.** GSEA figures for m6A-modified lncRNAs in HCC, LGG and GBM. A, F11-AS1 enriched in DNA repair pathway in HCC; B, LINC01018 enriched in DNA repair pathway in HCC; C, MIR325HG enriched in EMT pathway in LGG, D, MIR325HG enriched in EMT pathway in GBM.

**Additional file 3: Table S2.** List of m^6^A-regulated TE lncRNAs across tissues and cancers.

**Additional file 4: Table S3.** Expression correlation between m^6^A regulators and lncRNAs.

**Additional file 5: Table S4.** Clinical association of m^6^A-modified TE lncRNAs.

**Additional file 6: Table S5.** GSEA results for four m^6^A-modified TE lncRNAs.

## Data Availability

The gene expression profiles and clinical data can be found at the GDC portal (https://portal.gdc.cancer.gov/). The TE lncRNAs across tissues were obtained from LncSpA (http://bio-bigdata.hrbmu.edu.cn/LncSpA/). Software and resources used for the analyses are described in each method section. All results generated in this study can be found in supplementary tables.

## Abbreviations

m^6^A: methylation of N^6^ adenosine
GBM: Glioblastoma multiforme
LGG: Brain low grade glioma
lncRNA: Long non-coding RNA
TE: Tissue-elevated
LncSpA: LncRNA Spatial Atlas of expression
GSEA: Gene Set Enrichment Analysis
COAD: Colon adenocarcinoma
READ: Rectum adenocarcinoma
GTEx: The Genotype-Tissue Expression
HPA: Human Protein Atlas
HBM: Human body epigenome maps
FANTOM: Functional Annotation of the Mamaliam Genome

## References

[1] Wang S, Chai P, Jia R, Jia R (2018). Novel insights on m (6) a RNA methylation in tumorigenesis: a double-edged sword. Mol Cancer.

[2] Chen Y, Lin Y, Shu Y, He J, Gao W (2020). Interaction between N (6)-methyladenosine (m (6) a) modification and noncoding RNAs in cancer. Mol Cancer.

[3] Hon CC, Ramilowski JA, Harshbarger J, Bertin N, Rackham OJ, Gough J, Denisenko E, Schmeier S, Poulsen TM, Severin J, Lizio M, Kawaji H, Kasukawa T, Itoh M, Burroughs AM, Noma S, Djebali S, Alam T, Medvedeva YA, Testa AC, Lipovich L, Yip CW, Abugessaisa I, Mendez M, Hasegawa A, Tang D, Lassmann T, Heutink P, Babina M, Wells CA, Kojima S, Nakamura Y, Suzuki H, Daub CO, de Hoon MJ, Arner E, Hayashizaki Y, Carninci P, Forrest AR (2017). An atlas of human long non-coding RNAs with accurate 5′ ends. Nature.

[4] Lv D, Xu K, Jin X, Li J, Shi Y, Zhang M, Jin X, Li Y, Xu J, Li X (2020). LncSpA: LncRNA spatial atlas of expression across Normal and Cancer tissues. Cancer Res.

[5] Liu J, Li K, Cai J, Zhang M, Zhang X, Xiong X, Meng H, Xu X, Huang Z, Peng J, Fan J, Yi C (2020). Landscape and regulation of m (6) a and m (6) am Methylome across human and mouse tissues. Mol Cell.

[6] Li Y, Xiao J, Bai J, Tian Y, Qu Y, Chen X, Wang Q, Li X, Zhang Y, Xu J (2019). Molecular characterization and clinical relevance of m (6) a regulators across 33 cancer types. Mol Cancer.

[7] Chen S, Zhou L, Wang Y (2020). ALKBH5-mediated m (6) a demethylation of lncRNA PVT1 plays an oncogenic role in osteosarcoma. Cancer Cell Int.

[8] He Y, Hu H, Wang Y, Yuan H, Lu Z, Wu P, Liu D, Tian L, Yin J, Jiang K, Miao Y (2018). ALKBH5 inhibits pancreatic Cancer motility by decreasing long non-coding RNA KCNK15-AS1 methylation. Cell Physiol Biochem.

[9] Wang S, Xu M, Sun Z, Yu X, Deng Y, Chang H (2019). LINC01018 confers a novel tumor suppressor role in hepatocellular carcinoma through sponging microRNA-182-5p. Am J Physiol Gastrointest Liver Physiol.

[10] Deng Y, Wei Z, Huang M, Xu G, Wei W, Peng B, Nong S, Qin H (2020). Long non-coding RNA F11-AS1 inhibits HBV-related hepatocellular carcinoma progression by regulating NR1I3 via binding to microRNA-211-5p. J Cell Mol Med.

