# Supplementary material for: Pan-cancer characterization of expression and clinical relevance of m6A-related tissue-elevated long non-coding RNAs
Source: Mol Cancer. 2021 Feb 8;20:31. doi: 10.1186/s12943-021-01324-8 (PMC7869475; doi:10.1186/s12943-021-01324-8)
Supplement: Supplementary file 2 — Additional file 2: Supplemental materials and methods, and supplemental figure S1-S9.Figure S1. Numbers of m6A-regulated lncRNAs across tissues. Figure S2. Numbers of m6A-regulated TE lncRNAs across tissues in four data resources. Figure S3. Distribution of odds ratios for comparison between TE lncRNAs and non-TE lncRNAs across tissues in four data resources. Figure S4. Number of m6A peaks correlated with TE lncRNAs in four resources. Figure S5.Co-expression between m6A regulators and m6A-modified TE lncRNAs. A, River plot showing the expression correlation between m6A modified TE lncRNAs and m6A regulators. B, Bar plots showing the number of m6A regulators correlated with each m6A modified TE lncRNA. Color indicated the different data resources. C-E, Scatter plots showing the correlation between the expression of lncRNAs and m6A regulators. C for PVT1 and YTHDF2; D for SOX2-OT and HNRNPA2B1; E for KCNK15-AS1 and ALKBH5. Figure S6. Numbers of TE lncRNAs correlated with m6A regulators. Figure S7. Number of m6A modified TE lncRNAs across cancer types. Figure S8. GSEA for m6A modified lncRNAs in HCC, LGG and GBM. A, F11-AS1 in HCC; B, LINC01018 in HCC; C, MIR325HG in LGG, D, MIR325HG in GBM. Figure S9. GSEA figures for m6A-modified lncRNAs in HCC, LGG and GBM. A, F11-AS1 enriched in DNA repair pathway in HCC; B, LINC01018 enriched in DNA repair pathway in HCC; C, MIR325HG enriched in EMT pathway in LGG, D, MIR325HG enriched in EMT pathway in GBM. [file 12943_2021_1324_MOESM2_ESM.docx]

**Supplemental materials and methods**

**Tissue-elevated lncRNAs across tissues**

Long noncoding RNAs (LncRNAs) play important roles in maintaining morphology and function of tissues, whose regulatory effectiveness is closely associated with spatial expression. All the tissue-elevated lncRNAs (TE lncRNAs) were downloaded from LncSpA resource (<http://bio-bigdata.hrbmu.edu.cn/LncSpA>), which is a comprehensive spatial atlas of expression for lncRNAs [1]. In total, 71,131 and 12,007 TE lncRNAs were identified across 38 normal and 33 adult cancer tissues.

**Expression of protein-coding genes across tissues**

Four widely available transcriptome datasets were collected, including the Genotype-Tissue Expression (GTEx) consortium [2], Human BodyMap 2.0 (HBM2.0) [3], Human Protein Atlas (HPA) [4] and FANTOM5 project [5]. The gene expression profiles across 33 representing cancer types were generated by TCGA (http://cancergenome.nih.gov/), covering 11,093 samples. The clinical data of these cancer patients were also downloaded [6].

**m^6^A modification across tissues**

N6-methyladenosine (m^6^A) is the most abundant internal mRNA modification. We downloaded the m^6^A methylome across human tissues from one recent study, which included 43 human tissues [7]. The chromosome coordinates of m^6^A methylome peaks were downloaded from the supplemental files of this study.

**Identification of m^6^A regulated lncRNAs**

The chromosome coordinates of human lncRNAs were downloaded from GENCODE [8]. First, we manually mapped the tissues between m^6^A peaks and LncSpA. We next overlapped the lncRNA coordinates with m^6^A peaks. If an m^6^A peak was included in an lncRNA region, we defined this lncRNA was regulated by m^6^A modification. This process was performed by BEDTools [9].

**Tissue similarity based on m^6^A regulated lncRNAs**

To evaluate the similarity between tissues among different data resource, we calculated Simpson index between tissues based on lncRNAs with m^6^A modification. The similarity between tissue *i* and *j* was calculated as following:

$$S\left( i,j \right)=\frac{L_{i}\cap L_{j}}{min(L_{i},L_{j})}$$

where $L_{i}$ and $L_{j}$ were the m^6^A-related lncRNA sets in tissue *i* and *j*. The circular layout plot was plotted with R package circlize [10].

**Expression correlation between lncRNAs and m^6^A regulators**

We first collected the m^6^A regulators from one recent study [11]. Next, the gene expression correlation was calculated between the m^6^A regulators and TE lncRNAs. Pearson correlation coefficient (PCC) was calculated based on gene expression of m^6^A regulators and TE lncRNAs across tissues in different data resources. The m^6^A regulator-TE lncRNA pairs with p-value<0.05 were identified in each data resource.

**Differential expression of lncRNAs**

First, we select the cancer types with at least five adjacent tissues and also with m^6^A modified-TE lncRNA. Ten cancer types were identified for the following analysis. T-test and fold-change were calculated based on the lncRNA expression in cancer and normal samples of these cancers. LncRNAs with fold-change>2 or <0.5 and p-value<0.05 were considered as differentially expressed lncRNAs. Finally, we overlapped the differentially expressed lncRNAs and m^6^A modified-TE lncRNAs of the same cancer.

**Survival analysis based on lncRNA expression**

To identify the survival-related m^6^A-TE lncRNAs in cancer, we performed cox regression analysis and log-rank test for the cancer types with m^6^A modified-TE lncRNA. In total, 9 cancer types were screened. The univariate cox regression analysis was used to evaluate the association between survival and expression level of each m^6^A-TE lncRNA. In addition, all the patients were ranked based on the lncRNA expression. The top 20% and bottom 20% patients were defined as lncRNA high- or low-expressed groups. The differences in survival between the two groups were evaluated by the Kaplan–Meier method. Log-rank test was used to evaluate the survival significance. LncRNAs with p-values of log-rank test <0.05 were identified as survival-related lncRNAs. If patients with high expression of lncRNA were with significantly better survival, this lncRNA was defined as protective lncRNA. If patients with high expression of lncRNA were with significantly worse survival, this lncRNA was defined as risky lncRNA.

**Prediction of functions of lncRNAs**

To predict the function of m^6^A modified lncRNAs, we performed Gene Set Enrichment Analysis (GSEA) [12, 13]. First, we calculated the Pearson Correlation Coefficient (PCC) between the expressions of all protein coding genes with lncRNA of interest. The genes were ranked by the PCCs and subjected into pre-ranked GSEA. Here, we considered the functional sets associated with cancer hallmarks. Functional sets with p-adjusted<0.05 were considered as significant pathways.

**References**

1. Lv D, Xu K, Jin X, Li J, Shi Y, Zhang M, Jin X, Li Y, Xu J, Li X: **LncSpA: LncRNA Spatial Atlas of Expression across Normal and Cancer Tissues.** *Cancer Res* 2020, **80:**2067-2071.

2. Mele M, Ferreira PG, Reverter F, DeLuca DS, Monlong J, Sammeth M, Young TR, Goldmann JM, Pervouchine DD, Sullivan TJ, et al: **Human genomics. The human transcriptome across tissues and individuals.** *Science* 2015, **348:**660-665.

3. Klijn C, Durinck S, Stawiski EW, Haverty PM, Jiang Z, Liu H, Degenhardt J, Mayba O, Gnad F, Liu J, et al: **A comprehensive transcriptional portrait of human cancer cell lines.** *Nat Biotechnol* 2015, **33:**306-312.

4. Uhlen M, Oksvold P, Fagerberg L, Lundberg E, Jonasson K, Forsberg M, Zwahlen M, Kampf C, Wester K, Hober S, et al: **Towards a knowledge-based Human Protein Atlas.** *Nat Biotechnol* 2010, **28:**1248-1250.

5. Uhlen M, Hallstrom BM, Lindskog C, Mardinoglu A, Ponten F, Nielsen J: **Transcriptomics resources of human tissues and organs.** *Mol Syst Biol* 2016, **12:**862.

6. Hoadley KA, Yau C, Hinoue T, Wolf DM, Lazar AJ, Drill E, Shen R, Taylor AM, Cherniack AD, Thorsson V, et al: **Cell-of-Origin Patterns Dominate the Molecular Classification of 10,000 Tumors from 33 Types of Cancer.** *Cell* 2018, **173:**291-304 e296.

7. Liu J, Li K, Cai J, Zhang M, Zhang X, Xiong X, Meng H, Xu X, Huang Z, Peng J, et al: **Landscape and Regulation of m(6)A and m(6)Am Methylome across Human and Mouse Tissues.** *Mol Cell* 2020, **77:**426-440 e426.

8. Frankish A, Diekhans M, Ferreira AM, Johnson R, Jungreis I, Loveland J, Mudge JM, Sisu C, Wright J, Armstrong J, et al: **GENCODE reference annotation for the human and mouse genomes.** *Nucleic Acids Res* 2019, **47:**D766-D773.

9. Quinlan AR, Hall IM: **BEDTools: a flexible suite of utilities for comparing genomic features.** *Bioinformatics* 2010, **26:**841-842.

10. Gu Z, Gu L, Eils R, Schlesner M, Brors B: **circlize Implements and enhances circular visualization in R.** *Bioinformatics* 2014, **30:**2811-2812.

11. Li Y, Xiao J, Bai J, Tian Y, Qu Y, Chen X, Wang Q, Li X, Zhang Y, Xu J: **Molecular characterization and clinical relevance of m(6)A regulators across 33 cancer types.** *Mol Cancer* 2019, **18:**137.

12. Mootha VK, Lindgren CM, Eriksson KF, Subramanian A, Sihag S, Lehar J, Puigserver P, Carlsson E, Ridderstrale M, Laurila E, et al: **PGC-1alpha-responsive genes involved in oxidative phosphorylation are coordinately downregulated in human diabetes.** *Nat Genet* 2003, **34:**267-273.

13. Subramanian A, Tamayo P, Mootha VK, Mukherjee S, Ebert BL, Gillette MA, Paulovich A, Pomeroy SL, Golub TR, Lander ES, Mesirov JP: **Gene set enrichment analysis: a knowledge-based approach for interpreting genome-wide expression profiles.** *Proc Natl Acad Sci U S A* 2005, **102:**15545-15550.

**Supplemental figures**


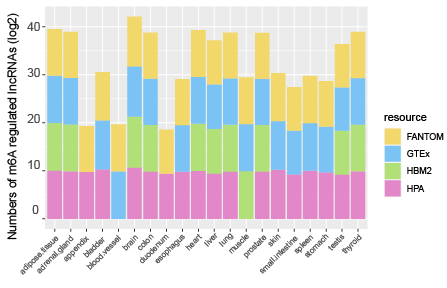


**Figure S1. Numbers of m^6^A-regulated lncRNAs across tissues.**


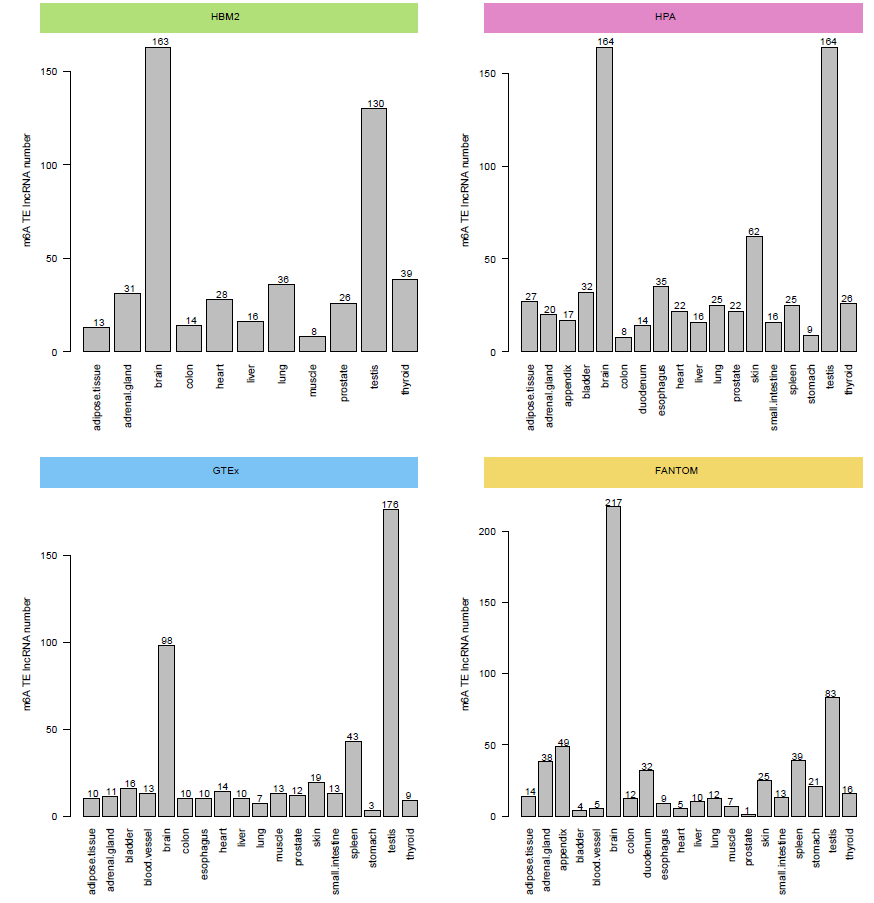


**Figure S2. Numbers of m^6^A-regulated TE lncRNAs across tissues in four data resources.**


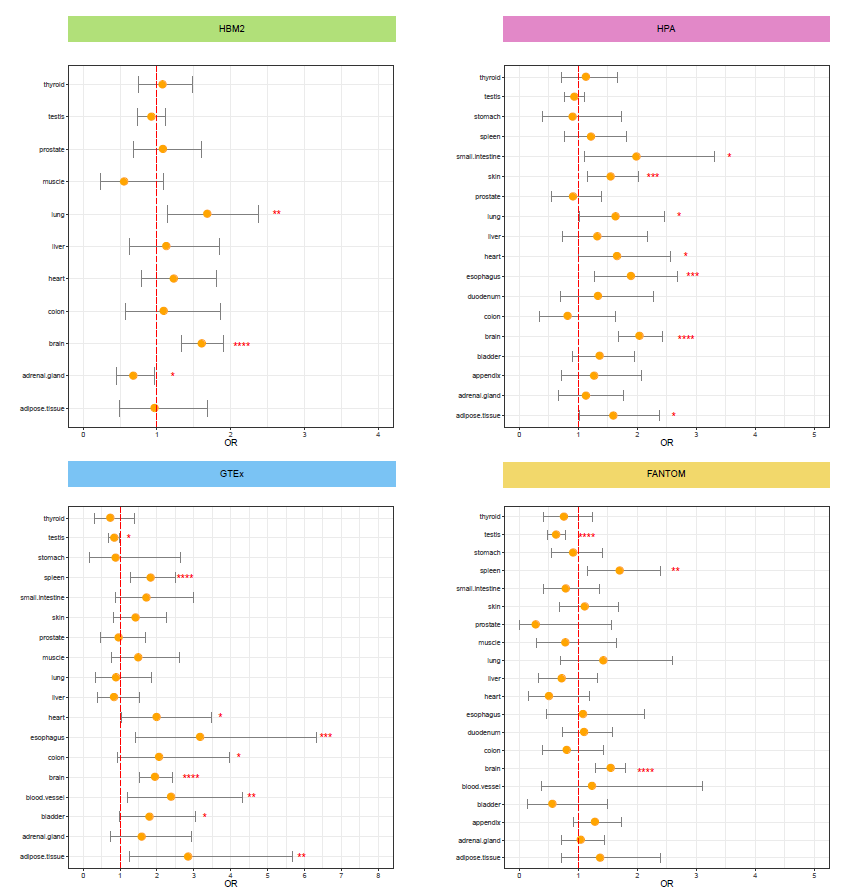


**Figure S3. Distribution of odds ratios for comparison between TE lncRNAs and non-TE lncRNAs across tissues in four data resources.**


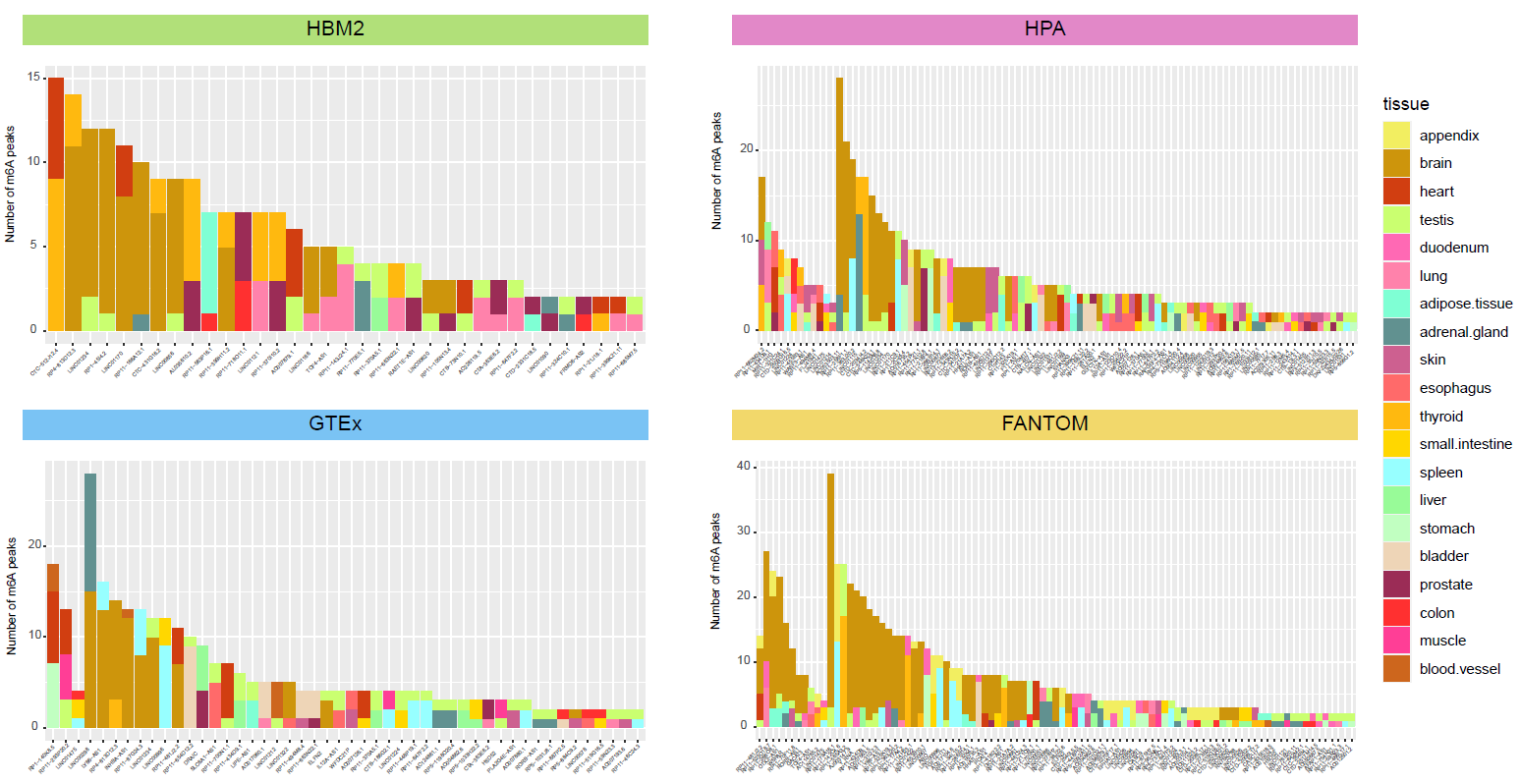


**Figure S4. Number of m^6^A peaks correlated with TE lncRNAs in four resources.**

**
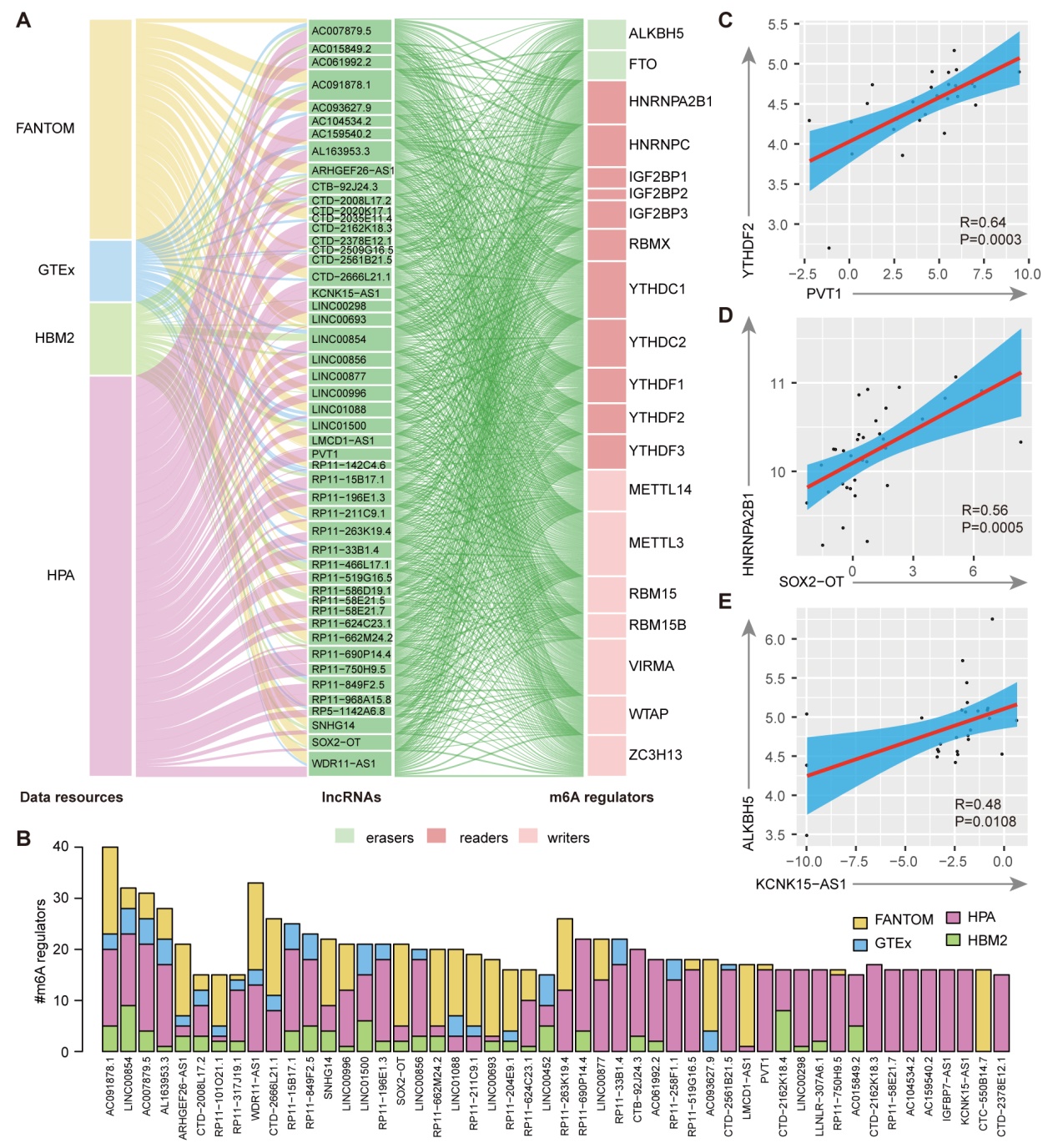
**

**Figure S5. Co-expression between m^6^A regulators and m^6^A-modified TE lncRNAs.** **A,** River plot showing the expression correlation between m^6^A-modified TE lncRNAs and m^6^A regulators. **B,** Bar plots showing the number of m^6^A regulators correlated with each m^6^A-modified TE lncRNA. Color indicated the different data resources. **C-E,** Scatter plots showing the correlation between the expression of lncRNAs and m^6^A regulators. **C** for PVT1 and YTHDF2; **D** for SOX2-OT and HNRNPA2B1; **E** for KCNK15-AS1 and ALKBH5.


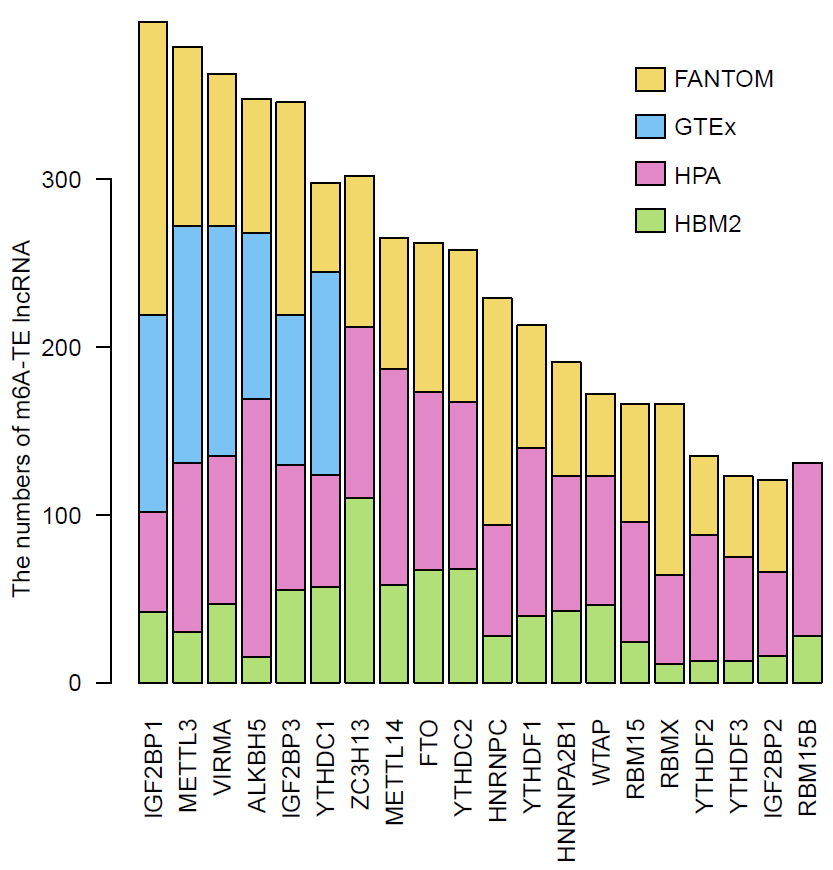


**Figure S6. Numbers of TE lncRNAs correlated with m^6^A regulators.**


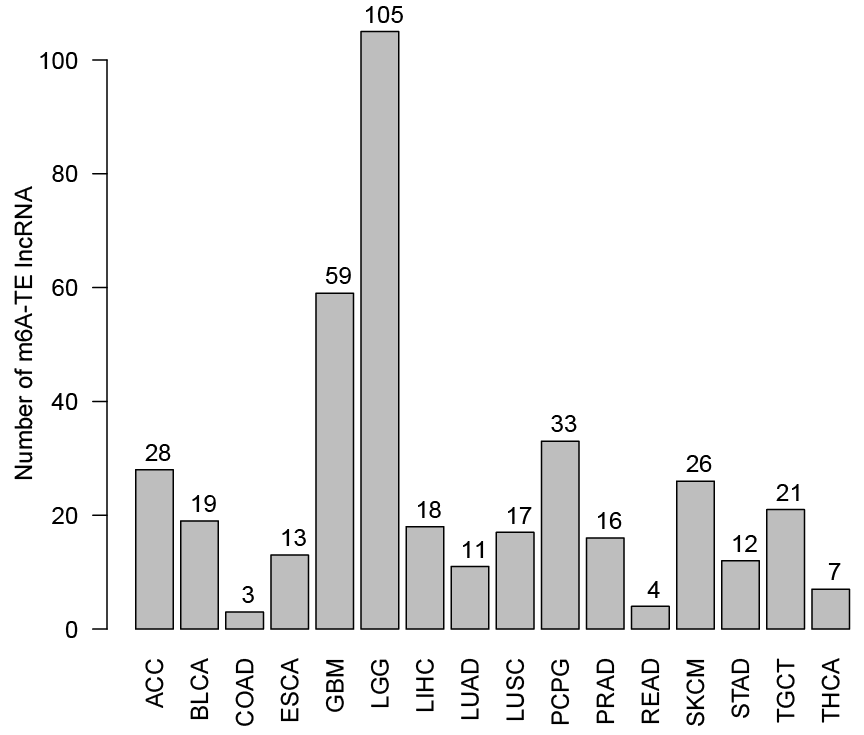


**Figure S7. Number of m^6^A-modified TE lncRNAs across cancer types.**

**
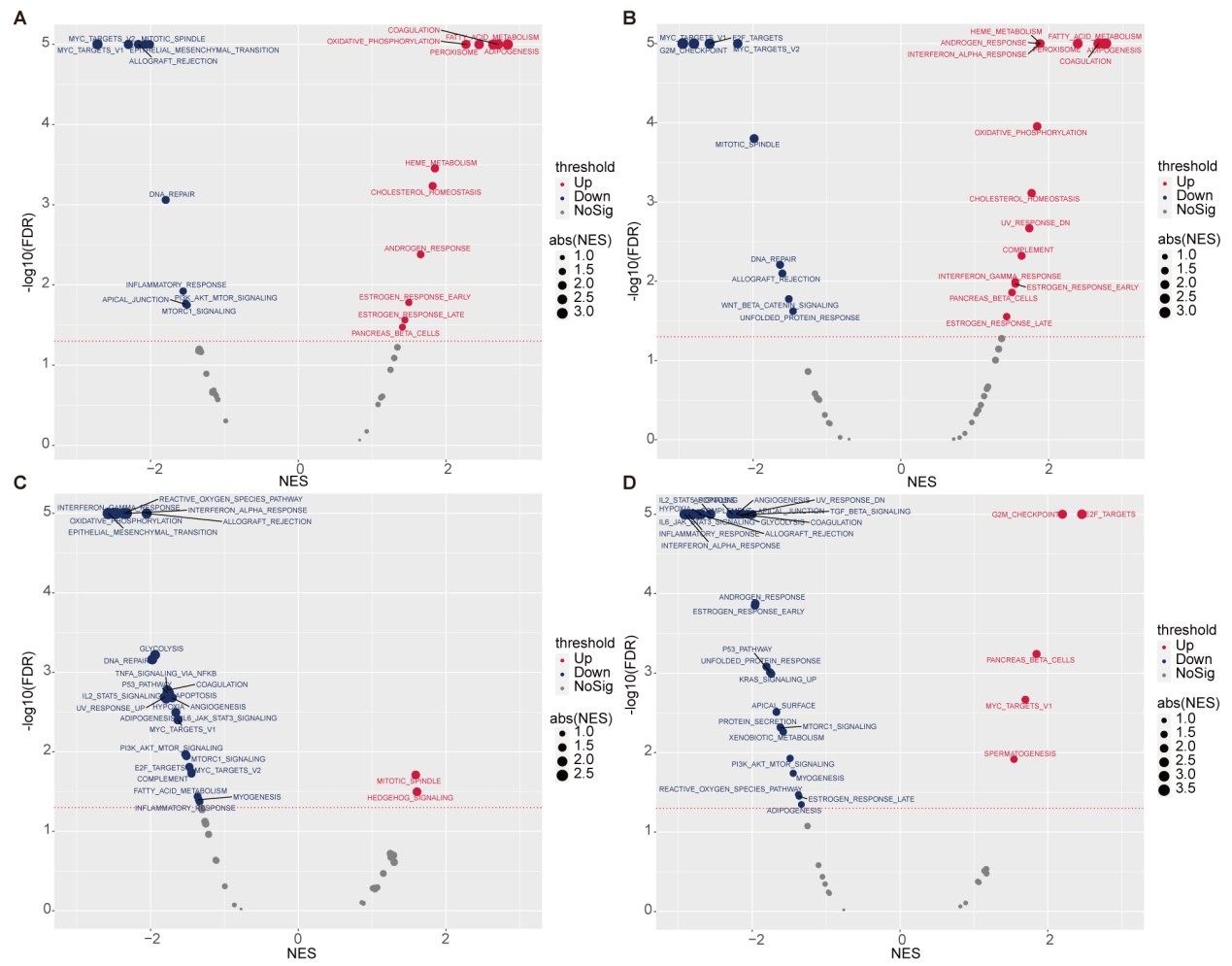
**

**Figure S8. GSEA for m^6^A-modified lncRNAs in HCC, LGG and GBM.**

A, F11-AS1 in HCC; B, LINC01018 in HCC; C, MIR325HG in LGG, D, MIR325HG in GBM.


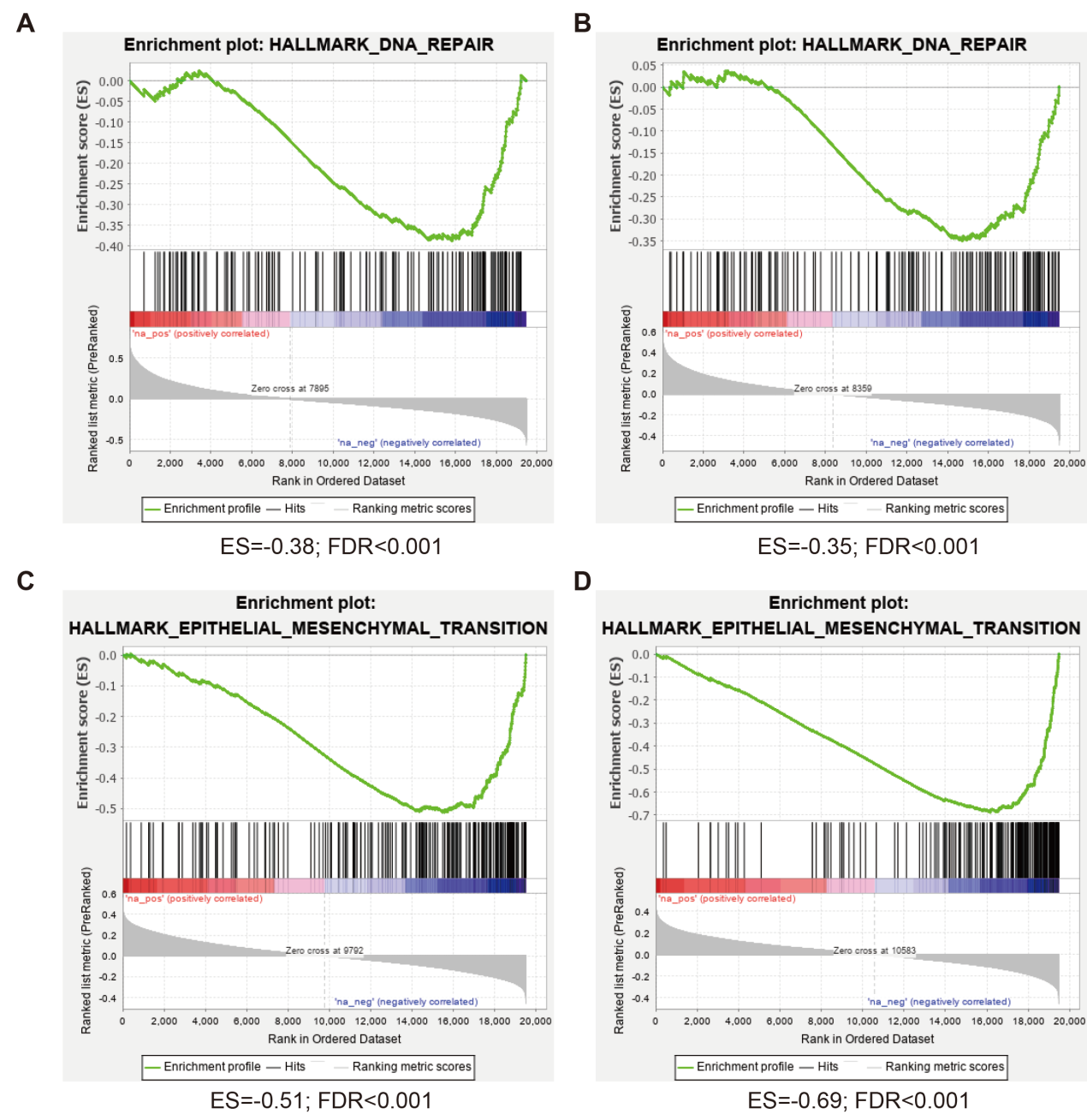


**Figure S9. GSEA figures for m^6^A-modified lncRNAs in HCC, LGG and GBM.**

A, F11-AS1 enriched in DNA repair pathway in HCC; B, LINC01018 enriched in DNA repair pathway in HCC; C, MIR325HG enriched in EMT pathway in LGG, D, MIR325HG enriched in EMT pathway in GBM.
